# Screening of cashmere fineness-related genes and their ceRNA network construction in cashmere goats

**DOI:** 10.1038/s41598-021-01203-8

**Published:** 2021-11-09

**Authors:** Taiyu Hui, Yuanyuan Zheng, Chang Yue, Yanru Wang, Zhixian Bai, Jiaming Sun, Weidong Cai, Xinjiang Zhang, Wenlin Bai, Zeying Wang

**Affiliations:** College of Animal Science & Veterinary Medicine, Shenyang Agricultural University, Shenyang, 110866 China

**Keywords:** Genetics, Animal breeding

## Abstract

Competitive endogenous RNA (ceRNA) is a transcript that can be mutually regulated at the post-transcriptional level by competing shared miRNAs. The ceRNA network connects the function of protein-encoded mRNA with the function of non-coding RNA, such as microRNA (miRNA), long non-coding RNA (lncRNA), and circular RNA (circRNA). However, compared with the ceRNA, the identification and combined analysis of lncRNAs, mRNAs, miRNAs, and circRNAs in the cashmere fineness have not been completed. Using RNA-seq technology, we first identified the miRNAs presented in Liaoning Cashmere Goat (LCG) skin, and then analyzed the mRNAs, lncRNAs, circRNAs expressed in LCG and Inner Mongolia cashmere goat (MCG) skin. As a result, 464 known and 45 new miRNAs were identified in LCG skin. In LCG and MCG skin, 1222 differentially expressed mRNAs were identified, 170 differentially expressed lncRNAs and 32 differentially expressed circRNAs were obtained. Then, qRT-PCR was used to confirm further the representative lncRNAs, mRNAs, circRNAs and miRNAs. In addition, miRanda predicted the relationships of ceRNA regulatory network among lncRNAs, circRNAs, miRNAs and mRNAs, the potential regulatory effects were investigated by Go and KEGG analysis. Through the screening and analysis of the results, the ceRNA network regulating cashmere fineness was constructed. LncRNA MSTRG14109.1 and circRNA452 were competed with miRNA-2330 to regulated the expression of TCHH, KRT35 and JUNB, which may provide a potential basis for further research on the process of regulating the cashmere fineness.

## Introduction

Liaoning Cashmere Goat is a goat breed with high-quality cashmere in China which originated in southeastern Liaoning Province. The variety has an advantage in producing cashmere and ranks first in the country. The cashmere goats of Inner Mongolia are mainly distributed in the northwest of Inner Mongolia Plateau, and their diameter is smaller than that of LCG, which takes advantage of this breed^1^. Wool collected from cashmere goats can be made into cashmere and clothing resisted the cold, which is in tremendous demand in the international market^2^. Later it was found that cashmere fineness was the main factor in the quality of cashmere. So far, the specific regulatory mechanism of cashmere fineness is not well realized. In order to explore further the key mechanism affecting cashmere fineness in cashmere goat skin. We studied and analyzed in two different types of cashmere fibers, searched for the key genes regulating cashmere fineness and the molecular mechanism regulating cashmere fineness expression. MiRNA is a type of non-coding RNA with a length of about 22 nt. It plays a key role in post-transcriptional gene regulation, and its goal is to target mRNAs to translational suppression and gene expression as an adaptor of the miRNA-induced silencing complex^3^. A single miRNA can simultaneously targets multiple target genes and influences the expression of signaling pathways involved in functional expression^4^. In the past, some miRNAs have been found in the skin of goats^5^. Some studies have found that miRNA interacts with target genes and lncRNA affects cashmere growth.

LncRNA is different classes of RNA molecules that are longer than 200 nucleotides^6^. Recent studies have shown that lncRNA regulates different levels of genes and has developmental and differentiation functions for organisms^7^. LncRNA can interacts with mRNA, miRNA and can arouset a number of different functions^8^. For example, lncRNA XLOC_008679 is involved in the regulation of cashmere fineness by targeting *KRT35*^9^. Furthermore, LncRNA MTC promotes fibroblast proliferation and regulates cashmere growth by activating NF-κB signaling pathway^10^.

Circular RNAs (circRNAs) are a novel type of long, non-coding RNAs^11^. It is a closed continuous loop distinct from linear RNA^12^. Studies suggest that circRNA may be involved in mammalian growth and differentiation and cancer^13^. In the past, few studies have shown that circRNA regulates the fineness of cashmere, nowadays, there is new evidence that circRNA may regulate the growth of cashmere^14^.

The molecular mechanism of ceRNA on regulating the growth and fineness of cashmere is unclear. Therefore, in order to understand the effect of ceRNA network on cashmere fineness, this study focus on the differential expression patterns of lncRNA, mRNA, and circRNA in the skin via high-throughput sequencing. Differentially expressed circRNAs, lncRNAs, and mRNAs were further confirmed using qRT-RCR. Subsequently, we constructed lncRNA–miRNA–mRNA, circRNA–miRNA–mRNA, lncRNA/circRNA–miRNA–mRNA ceRNA network in the skin of cashmere goats based on the interaction of lncRNA–miRNA, circRNA–miRNA and mRNA–miRNA. Our findings might provide new evidence for exploring the cashmere fineness regulation mechanism.

## Results

### Differential expression of lncRNAs, circRNAs, mRNAs and miRNAs

**Figure 1:**
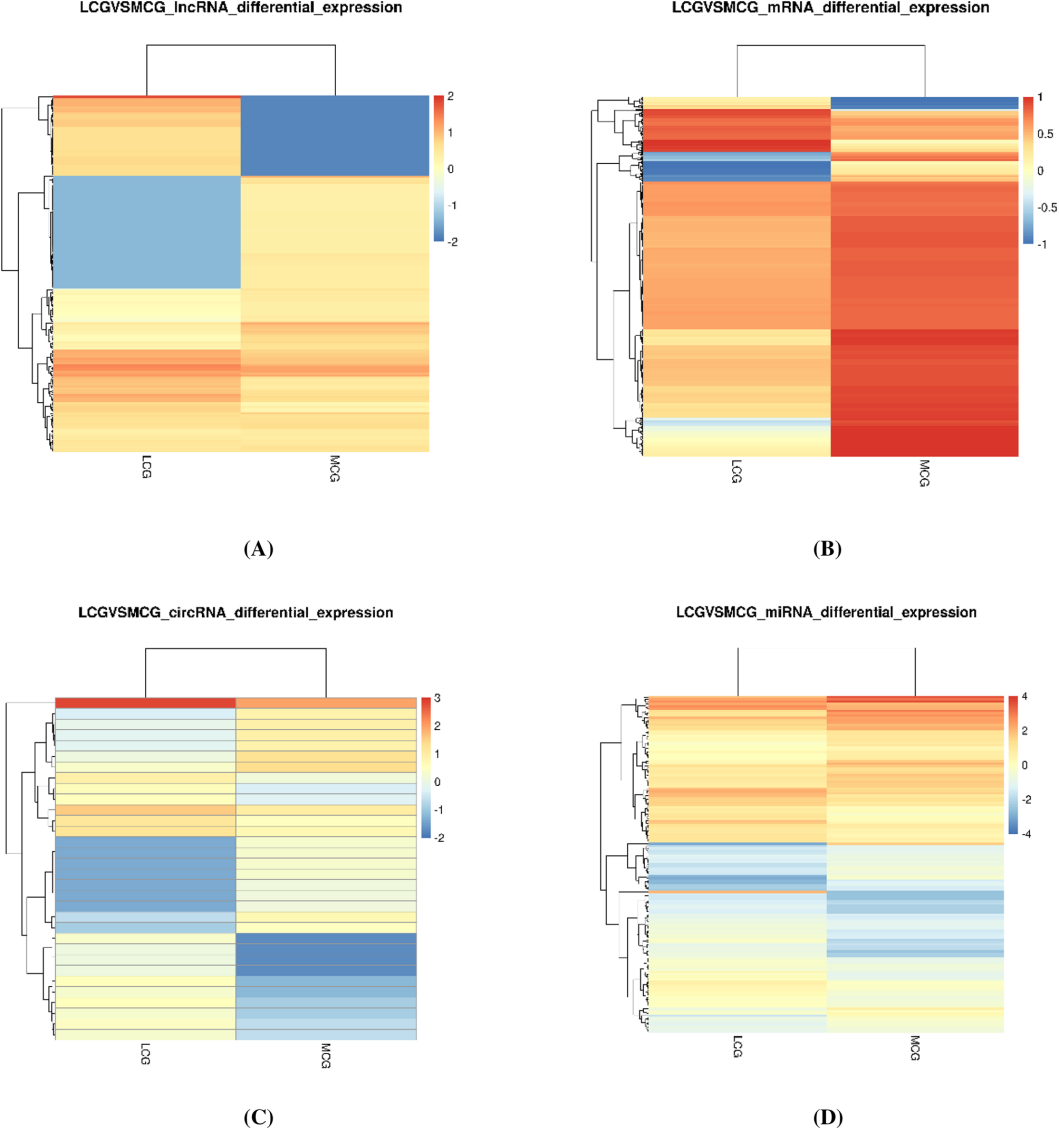
Heatmap of DElncRNAs, mRNAs, circRNAs and miRNAs between LCG and MCG. (**A**) Heatmap of DElncRNAs. (**B**) Heatmap of DEmRNAs. (**C**) Heatmap of DEcircRNAs. (**D**) Heatmap of DEmiRNAs.

**Figure 2:**
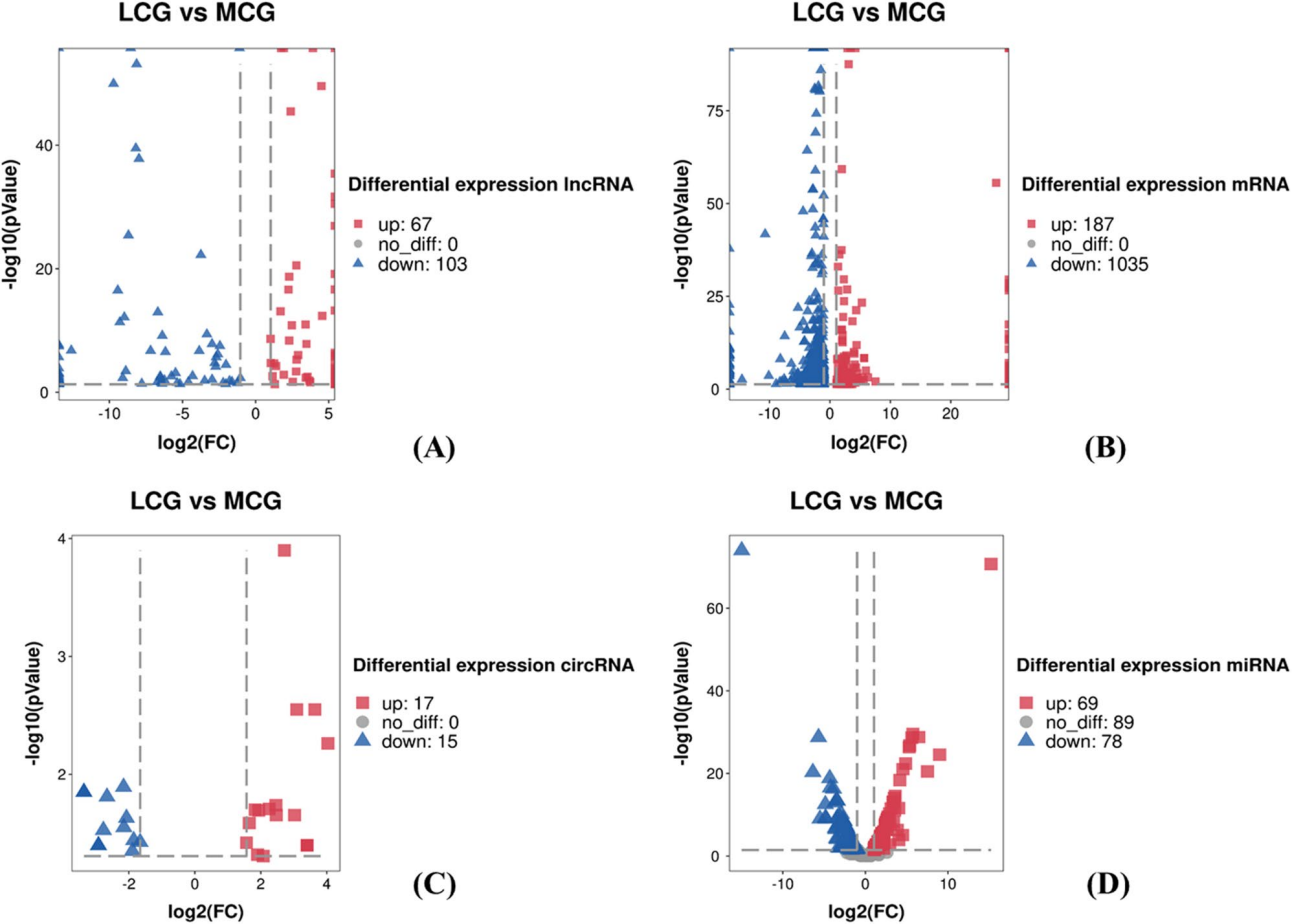
Volcano plot of DElncRNAs, mRNAs, circRNAs and miRNAs between LCG and MCG. (**A**) Volcano plot of DElncRNAs. (**B**) Volcano plot of DEmRNAs. (**C**) Volcano plot of DEcircRNAs. (**D**) Volcano plot of DEmiRNAs.

After the samples were extracted from the total RNA library for high throughput sequencing, we got 136,441,946 and 154,106,016 raw reads (Table 1). 128,989,956 and 146,038,848 valid data were obtained after removing adaptors and low-quality sequencing data. The screening conditions are q-value ≤ 0.05 and |log2FoldChange|≥ 1, it was found that a total of 1222 mRNAs were differentially expressed between the two groups of samples, including 187 up-regulated in LCG and 1035 down-regulated in LCG (Figs. 1, 2B).

**Table 1:** Sequence statistics and quality control of lncRNAs, circRNAs and mRNAs.

Sample	Raw reads	Valid reads	Q20%	Q30%	GC content%
LCG	136,441,946	128,989,956	98.72	89.48	50
MCG	154,106,016	146,038,848	99.01	91.89	46

170 lncRNAs were differentially expressed between the two groups of samples (Fig. 1A), including 67 up-regulating lncRNAs and 103 down-regulating in LCG (Fig. 2A). In up-regulation, MSTRG.5418.1, MSTRG.11385.1 showed slightly higher expression abundance and multiple than others, which may have important functions and are the main directions for follow-up studies.

A total of 32 circRNAs were differentially expressed (Fig. 1C), including 17 up-regulated in LCG and 15 down-regulated (Fig. 2C)^14^. Some genes can regulate multiple circRNAs, such as *TCHH*, which regulate four transcripts of ciRNA126, ciRNA127, ciRNA128, ciRNA129, through the analysis of differential expression of non-coding RNA. We collected skin samples and examined the expression of miRNA and small RNA in LCG (Fig. 1D). The sequencing results obtained an total of 15,245,657 raw reads (Table 2)^15^. Li et al. sequenced the miRNAs in the skin of MCG by high throughput^16^. After compared our data with theirs, 147 miRNAs with different expression were obtained, including 69 up-regulated miRNAs and 78 down-regulated miRNAs (q-value ≤ 0.05 and |log twofold change|≥ 1) (Fig. 2D).

**Table 2:** Sequence statistics and quality control of miRNAs.

Sample	Reads	Bases	Error rate (%)	Q20 (%)	Q30 (%)	GC content (%)
LCG	15,245,657	0.762G	0.01	97.98	93.08	49.24

### Differentially expressed lncRNAs/circRNAs/mRNAs/miRNAs validated by qRT-PCR

**Figure 3:**
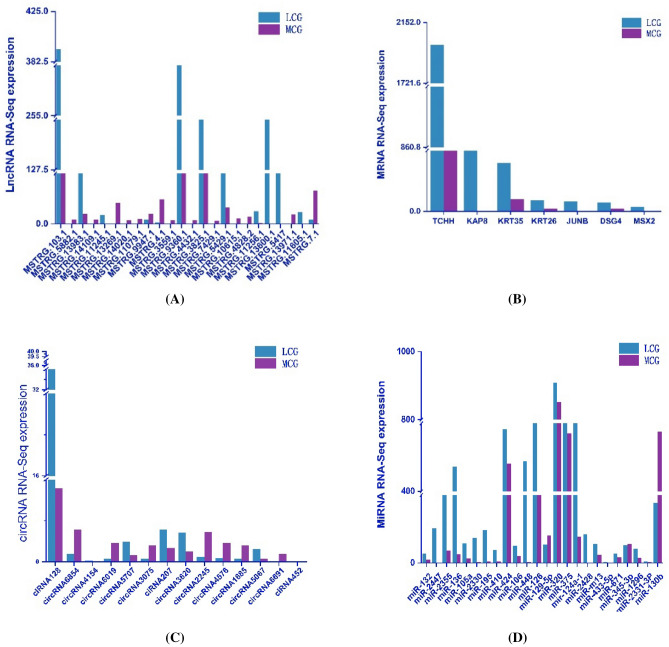
DElncRNAs, mRNAs, circRNAs and miRNAs between LCG and MCG in RNA sequencing (RNA-seq). (**A**) DElncRNAs between LCG and MCG in RNA-seq. (**B**) DEmRNAs between LCG and MCG in RNA-seq. (**C**) DEcircRNAs between LCG and MCG in RNA-seq. (**D**) DEmiRNAs between LCG and MCG in RNA-seq.

**Figure 4:**
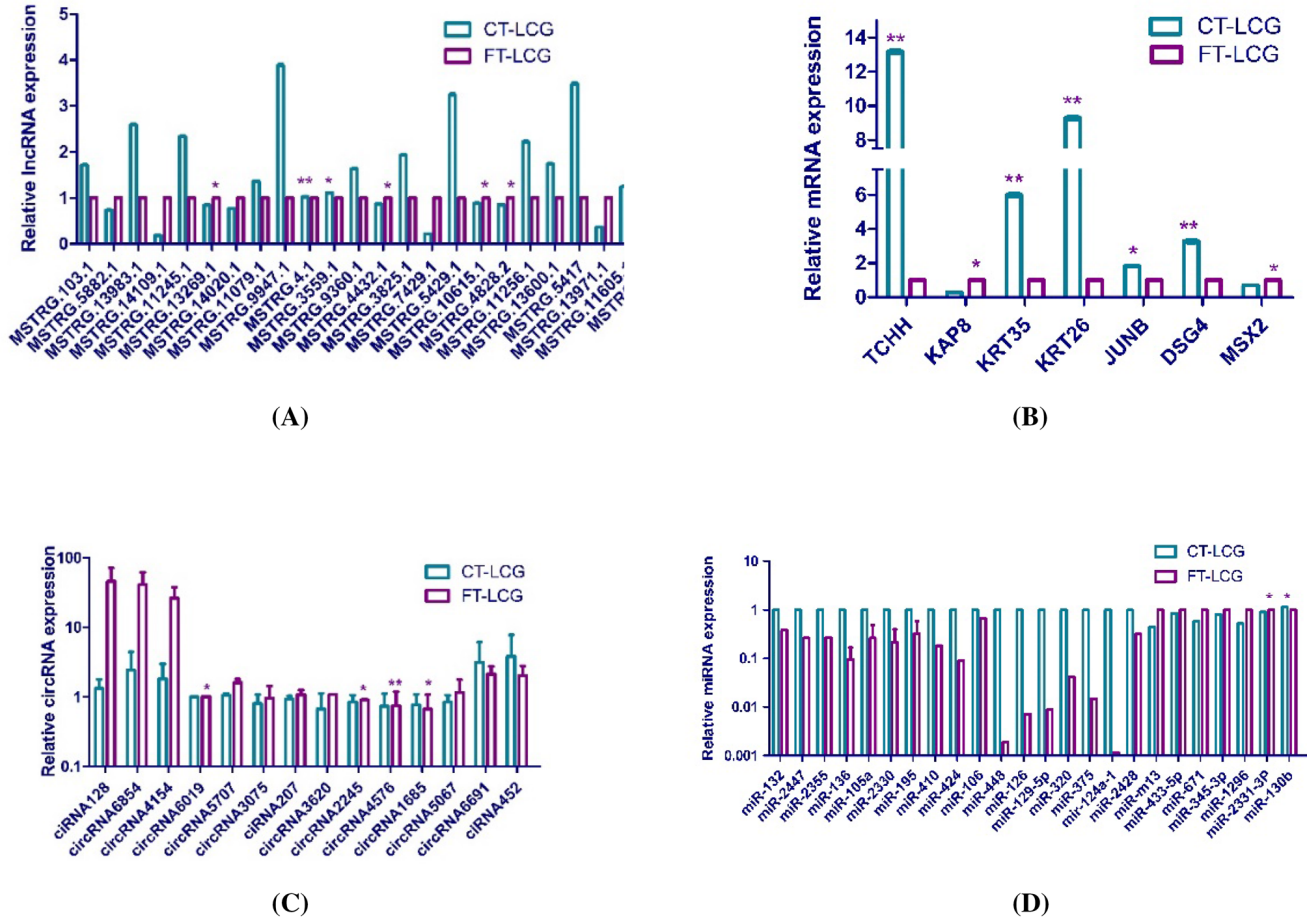
QRT-PCR verification of DElncRNAs, mRNAs, circRNAs and miRNAs between CT-LCG and FT-LCG. (**A**) DElncRNAs between CT-LCG and FT-LCG validated by qRT-PCR. (**B**) DEmRNAs between CT-LCG and FT-LCG validated by qRT-PCR. (**C**) DEcircRNAs between CT-LCG and FT-LCG validated by qRT-PCR. (**D**) DEmiRNAs between CT-LCG and FT-LCG validated by qRT-PCR.

To verify the results of the RNA-seq, we randomly selected 14 circRNAs, 24 lncRNAs, 24 miRNAs and 7 mRNAs in fine type LCG (FT-LCG) and coarse type LCG (CT-LCG) panels for qRT-PCR verification. The high-throughput results showed the difference between the diameters of the fine and the coarse types (Fig. 3). According to the 2^−(∆∆Ct)^ numerical values, it can be seen that the trend between the qRT-PCR results and the results of RNA-seq was consistent (Fig. 4). The expression level of FT-LCG is higher than that of CT-LCG, which may play a positive role in regulating candidate genes related to cashmere fineness. However, the expression level of CT-LCG is higher than that of FT-LCG, which may negatively regulate candidate genes related to cashmere fineness.

### Analysis between lncRNAs, circRNAs, miRNAs and mRNAs differential expression

**Figure 5:**
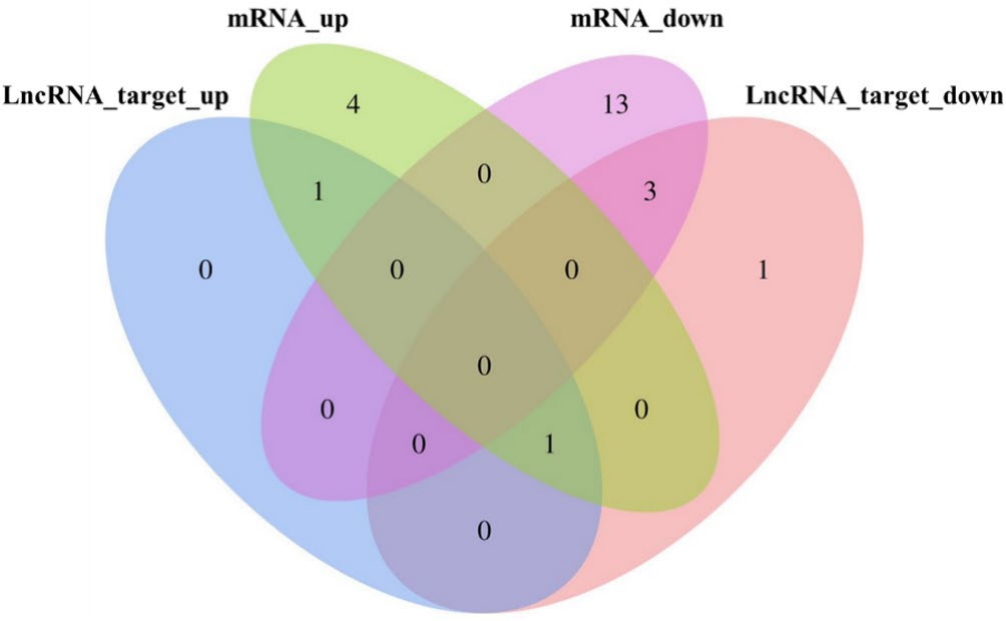
Veen gram showing the Integrative analysis of lncRNA targets and mRNAs.

We mainly performed predictive analysis of differential expression mRNAs, lncRNAs, miRNAs and circRNAs in RNA-seq results. By analyzing circRNA–mRNA, the results yield a key host gene, *TCHH*, which is simultaneously up-regulated in mRNA and circRNA. The 30 lncRNA–mRNA were significantly different, including 6 up-regulated mRNAs, 16 down-regulated mRNAs, 2 lncRNA target genes up-regulated, and 5 lncRNA target genes down-regulated. It was found that some key differentially expressed miRNAs can regulate related genes, such as miRNA-660-*KRT26*, miRNA-199a-3p-*DSG4*. MiRNA-125b, miRNA-19a (b), miRNA-339b, which may play a regulatory role in the target gene *KRT35*. MiRNA-141, miRNA-1940, miRNA-200a, miRNA-7 may be involved in the regulation of the target gene *JUNB*. Joint analysis found that the up-regulated mRNA *LOC108634775/XM_018045014.1* was both a target gene for up-regulated lncRNAs and a target gene for down-regulated lncRNAs (Fig. 5). Suggesting that a large number of genes may be regulated from different lncRNAs transcripts, whose expression are performed under the combined action of individual transcripts. Some of these genes are neither the up-regulated lncRNAs target gene nor the down-regulated lncRNAs target gene, indicating that this part of the gene expression may not be regulated by the relevant lncRNAs.

### Prediction of LncRNA-miRNA interactions

LncRNAs contain specific binding sequences of miRNAs, which can reduce their levels in the cytoplasm by adsorbing miRNAs to relieve the inhibition effect of miRNAs on target gene expression. In this section, the miRanda program was used to predict the targeted regulatory relationship between lncRNAs and miRNAs (Table 3). The results found that 458 interacted with miRNA-lncRNA.

**Table 3:** Interaction versus between lncRNA–miRNA.

lncRNA	miRNA	TargetScan_score	miRanda_Energy
MSTRG.11245.1	miR-185	96	− 22.24
MSTRG.13925.1	miR-2476	97	− 16.14
MSTRG.1626.1	miR-132	98	− 12.33
MSTRG.18.1	miR-193a-5p	97	− 21.61
MSTRG.3825.1	miR-320	97	− 19.13
MSTRG.3829.1	miR-2428	97	− 21.89
MSTRG.7654.1	miR-488	96	− 14.60
MSTRG.7899.1	miR-339	96	− 26.44
MSTRG.9911.1	miR-136	96	− 17.01
MSTRG.12784.1	miR-2411	97	− 16.74
MSTRG.4.1	miR-126	96	− 11.28
MSTRG.3970.1	miR-2447	97	− 29.56
MSTRG.10487.1	miR-9	96	− 12.69
MSTRG.10615.1	miR-1343	96	− 17.07
MSTRG.4383.1	miR-212	96	− 12.67
MSTRG.4383.1	miR-545	96	− 11.41

Table shows that the num of TargetScan fractional percentiles ≥ 96, − 30 < the num of Miranda maximum free energy values <  − 10.

### Prediction of circRNA–miRNA interactions

CircRNA can be used as an endogenous competing RNA by adsorbing miRNAs and some undiscovered ways to release the inhibition of miRNAs on their target genes, up-regulate the expression of target genes and then participate in the regulation of gene expression. The miRNAs and circRNAs were jointly analyzed by miRanda, resulting in 153 miRNA–circRNA relationship pairs, and finally 41 relationship pairs were screened out by the critical points predicted by software (Table 4).

**Table 4:** Interaction versus between circRNA–miRNA.

circRNA	miRNA	TargetScan_score	miRanda_Energy
ciRNA156	miR-2447	88	− 46.11
circRNA1685	miR-375	80	− 26.32
circRNA3620	miR-2331	80	− 17.68
circRNA5707	miR-136	80	− 18.74
circRNA6893	miR-9	80	− 10.02
circRNA815	miR-195	80	− 11.75
circRNA815	miR-424	80	− 31.55

Table shows that the num of TargetScan fractional percentiles ≥ 80, − 50 < the num of Miranda maximum free energy values <  − 10.

### Prediction of miRNA–mRNA interactions

MiRNAs can combine the target sequences of the untranslated ends of mRNAs to silence the mRNAs after transcription. The main mechanisms include promoting the degradation of mRNAs and inhibiting the translation of mRNAs. In order to better understand their potential functions, miRanda was used to analyze the targeted mRNAs of miRNAs. A total of 17,301 miRNA–mRNA relationship pairs were obtained, and 5605 relationship pairs were finally obtained through conditional screening (Table 5).

**Table 5:** Interaction versus between miRNA–mRNA.

mRNA	miRNA	TargetScan_score	miRanda_Energy
ACSL4	miR-375	99	− 10.69
IQGAP2	miR-545	99	− 10.04
LOC102179433	miR-2307	99	− 10.21
MRC1	miR-488	99	− 10.70
P2RY13	miR-375	99	− 10.66
PLXDC2	miR-375	99	− 10.48
SPATS2L	miR-105a	99	− 10.92
TMSB4X	miR-448	99	− 10.77

Table shows that the num of TargetScan fractional percentiles ≥ 99, − 11 < the num of Miranda maximum free energy values <  − 10.

### GO and KEGG pathway analyses of differentially-expressed mRNAs

**Figure 6:**
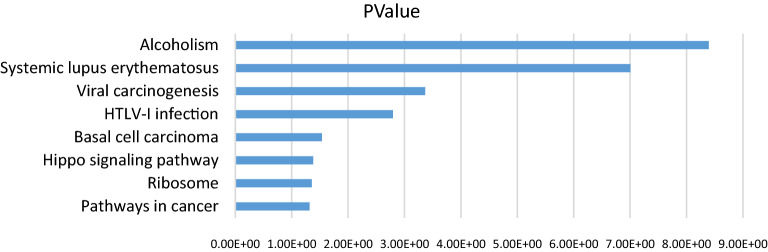
KEGG pathway analysis of 160 differentially expressed (DE)  mRNAs.

In the previous results, we predicted the target mRNA of miRNA based on miRanda, these target mRNAs are now compared with the differentially expressed mRNAs identified in the RNA-seq results to obtain 1062 differentially expressed mRNAs, of which 160 are up-regulated. To further understand the functional role of 160 different expression mRNAs (DEmRNAs) in cashmere fineness, we use DIVAD to analysis of GO and KEGG functional enrichment (Fig. 6). Based on the condition of p < 0.05, GO analysis is mainly significantly enriched in intermediate filament (CC) and structural molecule activity (MF) terms. The eight pathways enriched are shown as table, which may be key pathways to regulate the growth of cashmere fibers (Table 6).

**Table 6:** KEGG pathway analysis of 160 DE mRNAs.

Term	Pathway ID	Count	p value	Fold enrichment
Alcoholism	chx05034	11	4.02E−09	13.20952698
Systemic lupus erythematosus	chx05322	9	9.86E−08	14.5951417
Viral carcinogenesis	chx05203	7	4.30E−04	6.776315789
HTLV-I infection	chx05166	7	0.001605	5.270467836
Basal cell carcinoma	chx05217	3	0.028931	10.94635628
Hippo signaling pathway	chx04390	4	0.041647	5.026141513
Ribosome	chx03010	5	0.043986	3.620932101
Pathways in cancer	chx05200	6	0.048187	2.904135338

### Construction of lncRNA/circRNA-miRNA-mRNA network

**Figure 7:**
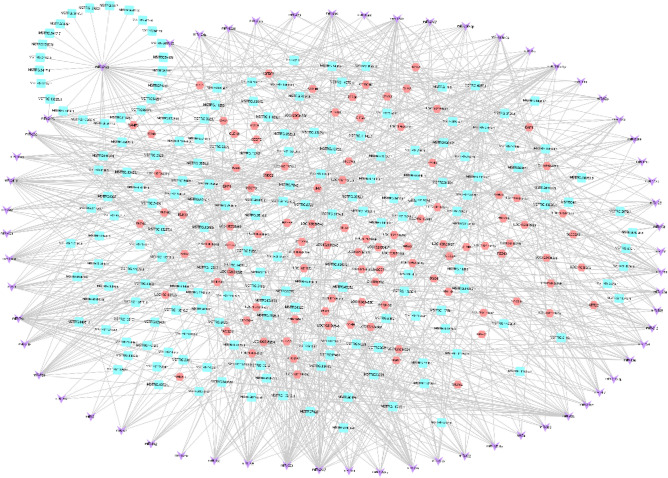
Regulation network of lncRNA–miRNA–mRNA. Pink: mRNAs, blue: lncRNAs, purple: miRNAs.

In this part, we selected 44,771 lncRNA–miRNA–mRNA interaction pairs. Considering that the total interaction network is too complex, it only demonstrates the expression regulation relationship with 160 up-regulated mRNAs as the core genes, which yielded 3060 lncRNA–miRNA–mRNA relation pairs, including 103 up-regulated mRNAs combined with 61 miRNAs and 195 lncRNAs. Construct a network regulation relationship of lncRNA–miRNA–mRNA with lncRNAs as decoy, miRNAs as core and mRNAs as target. Some key ceRNA networks were derived from the graph (Fig. 7), MSTRG.11079.1-miR-106-*DSG4*; MSTRG.4.1-miR-126-*TCHH*; MSTRG.14109.1 and miR-2330 co-regulate *JUNB*, *MSX2*, *KRT35* and *TCHH*.

**Figure 8:**
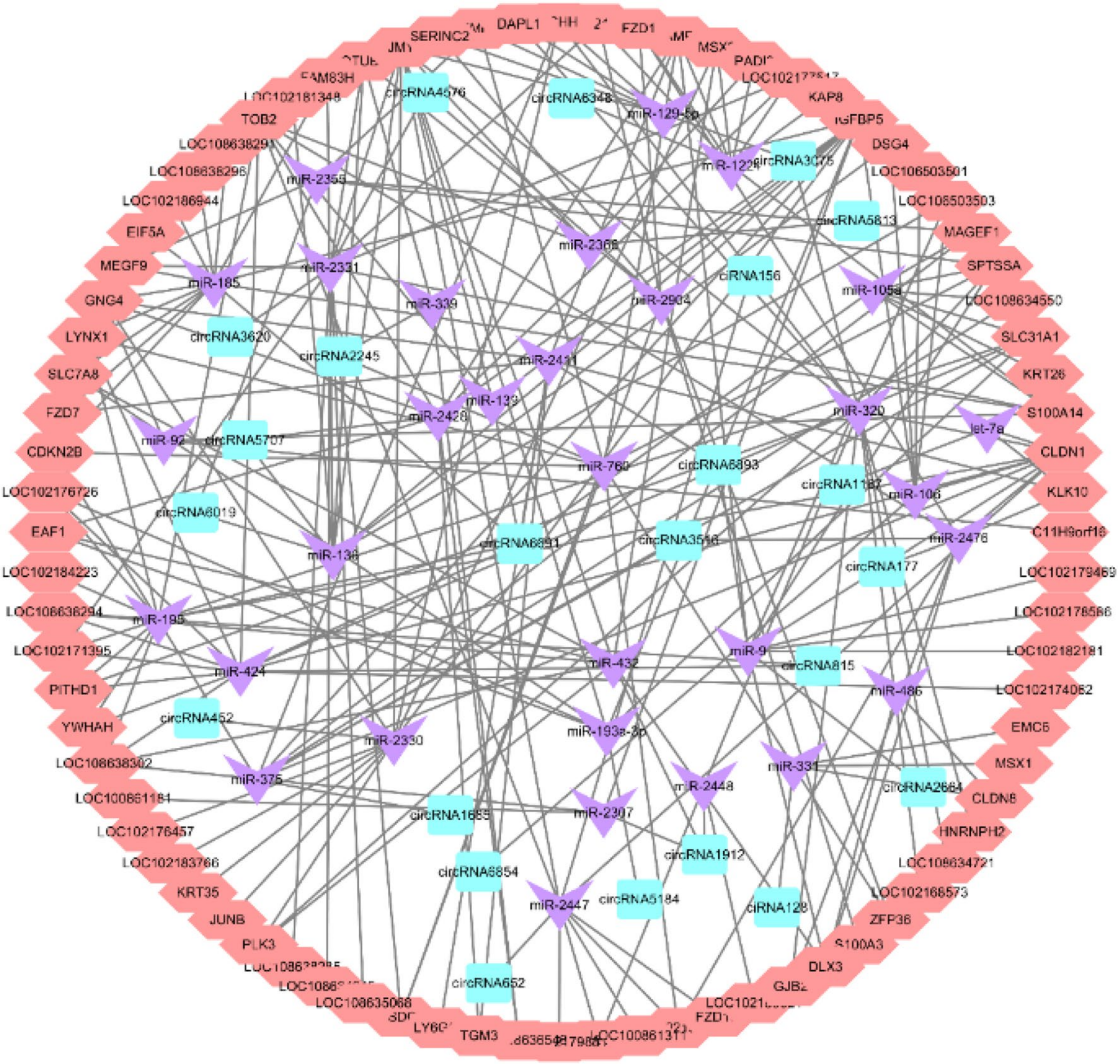
Regulation network of circRNA–miRNA–mRNA. Pink: mRNAs, blue: circRNAs, purple: miRNAs.

According to the theory of ceRNA and existing research, we find circRNA–mRNA interaction pairs with the same miRNAs binding site, and construct circRNA–miRNA–mRNA interaction pairs with circRNAs as decoy, miRNAs as core and mRNAs as targets. The predicted results showed that there were 4497 circRNA–miRNA–mRNA interaction pairs. Similarly, considering that all interaction networks are too complex, only 160 up-regulated mRNAs are shown as the core genes to bind the circRNAs and miRNAs, and 308 CircRNA–miRNA–mRNA relationship pairs are obtained, including 81 up-regulated mRNAs that bind to 32 miRNAs and 24 circRNAs (Fig. 8). The results showed that circ6854-miRNA-106-*DSG4*; circ3075-miRNA-129-5p-*TCHH*; circ452 and miRNA-2330 act simultaneously with *MSX2*, *TCHH*, *KRT35* and *JUNB*, which may be important regulatory pathways of cashmere fineness.

**Figure 9:**
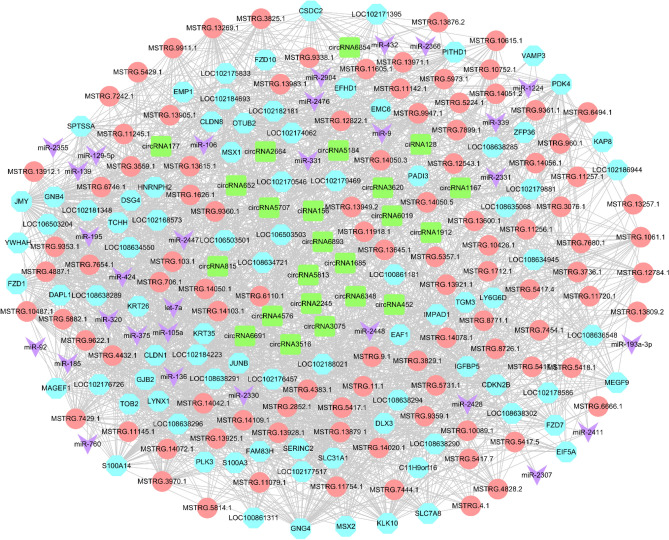
Regulation network of lncRNA/circRNA–miRNA–mRNA. Pink: lncRNAs, green: circRNAs, blue: mRNAs, purple: miRNAs.

**Figure 10:**
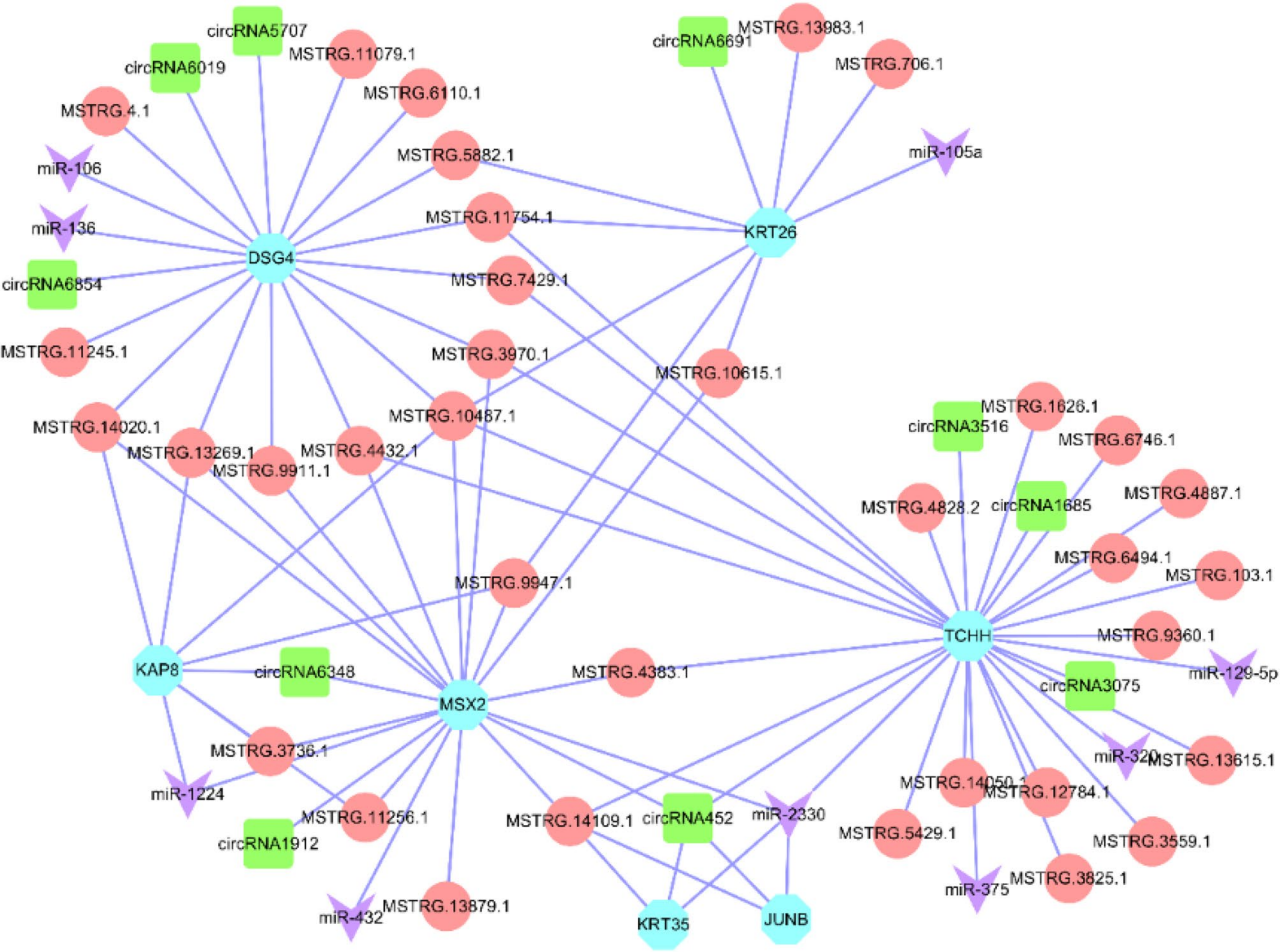
CeRNA networks of 7mRNAs, 35lncRNAs, 10circRNAs and 9miRNAs. Pink: lncRNAs, green: circRNAs, blue: mRNAs, purple: miRNAs.

For a deeper understanding of the interactions between lncRNAs, miRNAs, mRNAs, and circRNAs. The network of regulation relationship of lncRNA/circRNA–miRNA–mRNA was constructed under the interaction of lncRNA–miRNA, circRNA–miRNA and miRNA–mRNA (Fig. 9). The analysis shows that lncRNA-MSTRG.14020.1, lncRNA-MSTRG.11256.1, lncRNA-MSTRG.9947.1, lncRNA-MSTRG.13269.1, lncRNA-MSTRG.3736.1, lncRNA-MSTRG.10487.1 and circ6348 are ceRNA of miR-1224, and the target gene is *KAP8*. While lncRNA-MSTRG.706.1, lncRNA-MSTRG.9947.1, lncRNA-MSTRG.5882.1, lncRNA-MSTRG.13983.1, lncRNA-MSTRG.10487.1, lncRNA-MSTRG.11754.1, lncRNA-MSTRG.10615.1, and circ6691 are miR-105a targets ceRNA of *KRT26* (Fig. 10). Objective to construct the ceRNA regulatory network of non-coding RNA in cashmere goat skin and predict the network that may play a role in cashmere fineness growth.

## Discussion

Non-coding RNAs (ncRNAs) make up most of the transcribed genome and play different roles in numerous cellular processes. NcRNA includes such as miRNA, circRNA, and lncRNA^17^. Many studies have shown that these ncRNAs participate in competitive regulatory interactions, which is ceRNA networks^18^. Recently, studies have confirmed the significance of ceRNA in the regulation of skin diseases^19^ and hair follicle circulation^20^. The growth of cashmere is affected by many factors. The experimental samples are from modern standardized large-scale farms. With the same feeding standards and feeding environment, we want to analyze the differences in cashmere production from the genetic background. Based on the characteristics of LCG and MCG, we used these two breeds as high-throughput sequencing samples and constructs the regulatory relationship structure of the ceRNA network based on the correlation between ncRNAs. The difference of sequencing results indicated the difference of genetic material in different varieties. No research has been reported on the regulation of the ceRNA network related to LCG cashmere fiber growth thus far. Therefore, we first constructed a ceRNA regulatory network in cashmere goat skin.

We obtained maps of lncRNAs, circRNAs, mRNAs and miRNAs expression by high-throughput sequencing. The qRT-PCR results are basically consistent with the results of high-throughput analysis. In recent decades, the role of many genes in the regulation of cashmere goat growth has been widely studied. In recent years, researchers have reported that some genes can regulate cashmere fiber diameter through targeting relationships, such as *KRT6A*, *KRT38*, *KRT26*, *KRT32*, *KRT82*, *EGR3*, *FZD6*, *LncRNAMTC*, *KAP6.2*, *KAP7.1*, *KAP11.1*, *K26*, *BMPR_IB*, *LAMTOR3*, *KAP8.2* and *KAP26.1*, etc.^10,21,22^. In our study, 160 differentially expressed mRNAs were found by comparison. Among them, *MSX2*^23^, *KRT26*, *KRT35* and *KAP8*^24^ have been reported to affect cashmere growth and cashmere fineness in cashmere goats. *TCHH*^22,25^, *DSG4* expression in sheep skin^26^. *JUNB* can regulate epidermal stem cells and sebaceous glands by balancing the proliferation and differentiation of progenitor cells and suppressing lineage infidelity^27^. In mice, lack of *JUNB* wounds in skin delays healing and epidermal hyper proliferation^28^ and differential expression in the mammary glands of suckling goats^29^. However, most genes are regulated at the transcriptional level. Therefore, we speculated that these genes may have some role in regulating cashmere growth.

In order to further explore the key factors affecting the regulation of cashmere growth, we used miRanda and TargetScan to predict the relationship between lncRNA–miRNA, circRNA–miRNA, miRNA–mRNA. MiRNA, by pairing with bases of complementary sequences in mRNA molecules, can induce gene silencing by binding incomplete complementarity to mRNA 3ʹ-non-translational regions (3ʹ-UTRs)^30,31^. MiRNAs play an important role in gene regulation in plants and animals^32^. It has been reported that miRNA-mRNA interactions may affect cashmere growth. For example, the genes associated with cashmere growth in the Wnt signaling pathway are *FZD6*, *LEF1*, *FZD3*, *WNT5A*, and *TCF7*, and related miRNAs are miR-195, miR-148a, miR-4206, and recently, several groups have reported that gene products can regulate miRNA activity, and different RNA abundance values alter ceRNA interactions^33^.

LncRNA has multiple functions, including transcriptional activation, silencing of protein-coding genes, and interaction with mRNA or miRNA to regulate its function. Studies indicate that lncRNA-HOTAIR transcripts may be related to the formation and growth of cashmere fiber^34^. In recent years, lncRNA has become a previously undiscovered gene expression regulator that can regulate various cellular processes. Related research reports lncRNA LNC_000972, LNC_000503 and LNC_000881 may regulate hair follicle circulation through genes of *WNT3A*, *HOXC13* and *MSX2*^35^. Increasing evidence suggests that the effect of lncRNA on mRNA may regulate its function. Studies have found that the relative expression of *ET-1*, *SCF*, *ALP* and *LEF1* in dermal papilla cells can be increased by increasing the expression of lncRNA-000133, which may be related to the formation and growth of cashmere fibers^36^. Later research found that lncRNA can affect cashmere growth through mutual regulation with target genes^37^.

The covalently enclosed circRNA is produced by back-splicing the precursors of the exons of thousands of genes in eukaryotes^38^, which makes the resistance to nucleic acid exonuclease enhanced and therefore stable^39^. Emerging evidence suggests that circRNA a key role in cancer^40^, cell growth and differentiation^41^. In the Steroid-induced osteonecrosis of the femoral head-bone marrow mesenchymal stem cells, five circRNA targeted mRNAs including *CLASP1*, *ENOX2*, *SYK*, *UBE2G2*, and *WNT5B* were up-regulated by circRNA^42^. It has been proven in animals that it can be regulated by establishing a network of dynamic regulatory relationships between circRNA and mRNA^43^. The three circRNAs (chi-circ-1926, chi-circ-2829, and chi-circ-0483) and their corresponding host genes (*BNC2*, *PAPPA*, and *ZNF638*) may have certain functional roles in the development of cashmere fiber growth by constituting perfect regulatory pairs^44^.

Here, we build the lncRNA/circRNA–miRNA–mRNA ceRNA network based on our data for the first time. Nevertheless, the activity of some miRNAs has been shown to be mediated by lncRNA, further reducing the number of miRNA able to bind to mRNA target activity by separating the said activity^45^. Research indicates that the lncRNA–miRNA–mRNA regulatory network may play a role in the growth of cashmere fibers. For instance, the putative lncRNA-599554 may regulate the expression of potential target genes by regulating the expression of four miRNAs (miR-548d-3p, miR-15a-5p, miR-497-5p and miR-424-5p), such as *AXIN2*, *BTRC*, *CCND1* and *WNT5A*, miR-17-5p target two putative lncRNAs: lncRNA-599618 and lncRNA-599556, which are also involved in the regulation of *MYC*, *CCND1*, *CCND2*, *MAPK9* and *SMAD4* genes^37^. Then, the ceRNA network miR-221-5p-lnc_000679-*WNT3*, miR-34a-lnc_000181-*GATA3*, and miR-214-3p-lnc_000344-*SMAD3* have regulatory roles in hair follicle biology of cashmere goats^35^. Therefore, it is clear that the lncRNA–miRNA–mRNA interaction ultimately regulates the expression pattern of the target gene.

In recent years, because circRNA contains miRNA response elements and competes with mRNA, the potential of circRNA as a gene regulator has shown^46^. CircRNA inhibits the function of miRNA as a miRNA sponge through a competitive endogenous RNA (ceRNA) network, and then regulates mRNA expression through the intended target site of the miRNA^47^. Some studies have identified a potential target for regulating receptive endometrium development by regulating the expression of circRNA-9119-miR-26a-*PTGS2* in endometrial epithelium cells^48^. It was pointed out that circular RNA may play an important role in the secondary hair follicles of cashmere goats through the circRNA–miRNA–mRNA regulatory network, which explained the molecular mechanism of secondary hair follicle development and cashmere fiber growth in cashmere goats^44^.

According to the ceRNA network, some reports show that lncRNA H19 and circRNA MYLK can compete with miRNA-29a-3p and to increase the expression of the target genes *DNMT3B*, *VEGFA* and *ITGB1* to regulate bladder cancer^49^. In mice, lncRNA Meg3 and circRNA Igf1r can compete with miRNA-15a-5p to increase the expression of target genes *Inha*, *Acsl3*, *Kif21b*, *Igfbp2* and participate in the development of germ cells^50^. Results show some specific cashmere growth and fineness related ceRNA networks involved 36 lncRNAs (MSTRG.14109.1, MSTRG.9947.1, MSTRG.13269.1, etc.), 10 circRNAs (circ6854, circ5707, circ452, etc.), 9 miRNAs (miR-2330, miR-105a, miR-375, miR-129-5p, miR-106, etc.) and 7 mRNAs (*MSX2*, *KRT26*, *TCHH*, *KAP8*, *KRT35*, *JUNB*, *DSG4*). For example, lncRNA-MSTRG.14109.1, circ452 can regulate the expression of *KRT35* and *JUNB* through miR-2330, thereby affecting cashmere growth and fineness. These data indicate that construction ceRNA regulation network plays an important regulatory role in cashmere growth and fineness and may enrich the understanding of cashmere growth and fineness regulation and lay a foundation for future research.

In the current research, based on the sequencing data of lncRNAs, circRNAs and miRNAs, we constructed the ceRNA network through the joint correlation analysis of lncRNAs, circRNAs, mRNAs and miRNAs. It should be noted that this study only focuses on the two species of LCG and MCG, and our results lack further experimental verification. Despite its limitations, this study does show that some key ceRNA pathways may play a regulatory role in the regulation of cashmere fiber diameter, which may provide some ideas for the unknown regulation mechanism of cashmere fiber.

In conclusion, the transcriptome of two types of cashmere goat skin was studied by high-throughput sequencing. The results showed that there were differences between fine type (MCG) and coarse type (LCG) breeds. QRT-PCR verified the difference between fine and coarse breeds showed there was a consistent trend of gene and expression regulation between and within breeds. *MSX2*, *KRT26*, *KRT35*, *TCHH*, *KAP8*, *JUNB* and *DSG4* are likely in regulating cashmere growth and fineness. LncRNA MSTRG14109.1 and circRNA452 may compete with miRNA-2330 to regulate the expression of the target genes *TCHH*, *KRT35* and *JUNB*. Nevertheless, these may provide new insights for future research on cashmere growth and regulation of cashmere fineness.

## Materials and methods

### Ethics statement

All experimental protocols were approved by the Laboratory Animal Management Committee of Shenyang Agricultural University (No. 201806011, Shenyang, China). All methods were carried out in accordance with relevant guidelines and regulations. The study was carried out in compliance with the ARRIVE guidelines.

### Sample processing

We collected each samples of three adult female Liaoning cashmere goat scapular skins of Animal Husbandry Science Liaoning Province and three adult female Inner Mongolia cashmere goat scapular skins of Inner Mongolia Hohhot and Ringer Cashmere Goat Breeding Farm for high-throughput sequencing of miRNA, lncRNA, mRNA and circRNA. They are all 1.5-year-old females during anagen and in the same growth environment and fed the same feed. Fiber diameter of three adult female LCG skin samples (d = 19.4 µm, 19.5 µm and 19.8 µm), there adult female MCG (d = 13.8 µm, 14.0 µm and 14.1 µm) 0.1 cm^2^ of scapular skin after anesthesia following animal welfare be stored in liquid nitrogen immediately after cleaning until RNA separation. In addition, three coarse and fine skins of Liaoning cashmere goats were collected for high-throughput sequencing of miRNA and qRT-PCR verification. Three LCG (CT-LCG) skin fiber diameter (d = 19.5 µm, 19.7 µm and 20.2 µm), three LCG (FT-LCG) skin fiber diameter (d = 15.3 µm, 15.4 µm and 15.6 µm).

### Experimental procedure

To extract DNA from samples according to Takara kit instructions (Invitrogen, USA). Next, NanoDrop (NanoDrop Technologies, USA) determination RNA purity. Evaluation of RNA integrity using the internationally recognized Agilent 2100 (agilent technologies, USA) and the molar concentration was calculated. Ribosomal RNA consumption is about 5 ug of total RNA. Two sample libraries were constructed with NEBNext (Illumina Corporation, USA) after total RNA extraction. IlluminaHiseq4000 is finally used for sequencing, and the length of both ends of the sequencing read is 2 × 150 bp (pe150).

### Bioinformatics process

Firstly, Cutadapt^51^ was used to remove the reads that contained adaptor contamination, low quality bases and undetermined bases. Then sequence quality was verified using FastQC (http://www.bioinformatics.babraham.ac.uk/projects/fastqc/). We used Bowtie2^52^ and Tophat2^53^ to map reads to the genome of *Capra hircus*. Remaining reads (unmapped reads) were still mapped to genome using tophat-fusion^54^.

CIRCExplorer^55,56^ was used to denovo assemble the mapped reads to circular RNAs at first; Then, back splicing reads were identified in unmapped reads by tophat-fusion and CIRCExplorer. All samples were generated unique circular RNAs. The differentially expressed circRNAs were selected with log2 (fold change) > 1 or log2 (fold change) <  − 1 and with statistical significance (p value < 0.05) by R package–edgeR^57^.

The mapped reads of each sample were assembled using StringTie^58^. Then, all transcriptomes from *Capra hircus* Samples were merged to reconstruct a comprehensive transcriptome using perl scripts. After the final transcriptome was generated, StringTie^58^ and Ballgown^59^ was used to estimate the expression levels of all transcripts.

### Differential expression of lncRNAs, circRNAs, mRNAs and miRNAs

Analysis of our sequencing results with data from Liu et al. using R language yielded differentially expressed miRNAs in LCG and MCG. First of all, transcripts that overlapped with known mRNAs and transcripts shorter than 200 bp were discarded. Then we utilized CPC^60^ and CNCI^61^ to predict transcripts with coding potential. All transcripts with CPC score <  − 1 and CNCI score < 0 were removed. The remaining transcripts were considered as lncRNAs. StringTie^58^ was used to perform expression level for mRNAs and lncRNAs by calculating FPKM^62^. The differentially expressed mRNAs and lncRNAs were selected with |log2FoldChange|≥ 1 by R package—Ballgown^59^.

Secondly, Cutadapt^51^ was used to remove the reads that contained adaptor contamination, low quality bases and undetermined bases. Then sequence quality was verified using by FastQC (http://www.bioinformatics.babraham.ac.uk/projects/fastqc/). We used Bowtie2^52^ and Tophat2^53^ to map reads to the genome of species. Remaining reads (unmapped reads) were still mapped to genome using tophat-fusion^54^. CIRCExplorer^55,56^ was used to denovo assemble the mapped reads to circular RNAs at first; Then, back splicing reads were identified in unmapped reads by tophat-fusion and CIRC Explorer. All samples were generated unique circular RNAs. The differentially expressed circRNAs were selected with log2 (fold change) > 1 or log2 (fold change) <  − 1 and with statistical significance (p value < 0.05) by R package–edgeR^57^.

Finally, for the samples with biological replicates: Differential expression analysis of two conditions/groups was performed using the DESeq R package (1.8.3). The p-values was adjusted using the Benjamini and Hochberg method. Corrected p-value of 0.05 was set as the threshold for significantly differential expression by default. For the samples without biological replicates: Differential expression analysis of two samples was performed using the DEGseq (2010) R package. p-value was adjusted using qvalue^63^. q-value ≤ 0.05 and |log 2 Fold Change|≥ 1 was set as the threshold for significantly differential expression miRNAs by default.

### Quantitative real-time PCR

We selected 14 circRNAs, 24 lncRNAs, 24 miRNAs and 7 mRNAs for qRT-PCR verification. Total RNA was isolated from the skin of two groups of samples, each 1 μg of RNA samples were reverse transcribed into cDNA using cDNA, cDNA reverse transcription kit (Takara, Dalian, China). Primer 5.0 was used to design primers for quantitative PCR. Three CT-LCG and three FT-LCG skin samples were subjected to independent experiments and repeated three times each. The *GAPDH* gene was used as an internal control to normalize the expression level of the gene. Graph Pad Prism 5 software was used to display the 2−△△CT method to analyze expressed gene data. Using SPSS for biometric analysis, the difference was considered significant when p < 0.05 (*p < 0.05; **p < 0.001).

### Functional analysis

We predicted target mRNAs based on miRanda’s miRNAs, and these target mRNAs are now compared to the differentially expressed mRNAs identified in the RNA-seq results to obtain the intersecting elements of the target mRNA and the differentially expressed mRNAs. DIVAD software was then used to predict the GO function annotation and KEGG^64^ pathway of the intersecting mRNA to analyze its potential function.

### ceRNA network construction and bioinformatics analysis

The interactions of lncRNA–miRNA, circRNA–miRNA and mRNA–miRNA were predicted by using TargetScan7.0 and miRanda software. TargetScan 7.0 predicts miRNA targets based on the homology of the seed region^65^, while miRanda is mainly based on the combination of free energy generated by miRNAs in combination with their target genes^66^. The lower the free energy, the stronger the binding. TargetScan has hundreds of quantiles ≥ 50, and Miranda’s maximum free energy value <  − 10 and Miranda score > 140 are defined as the critical points for target prediction. Based on the data of lncRNA–miRNA, circRNA–miRNA and mRNA–miRNA, Cytoscape 3.6.0 software constructed lncRNA–miRNA–mRNA, circRNA–miRNA–mRNA, lncRNA/circRNA–miRNA–mRNA ceRNA network.
